# Food Combinations in Relation to the Quality of Overall Diet and Individual Meals in Japanese Adults: A Nationwide Study

**DOI:** 10.3390/nu12020327

**Published:** 2020-01-26

**Authors:** Kentaro Murakami, M. Barbara E. Livingstone, Nana Shinozaki, Minami Sugimoto, Aya Fujiwara, Shizuko Masayasu, Satoshi Sasaki

**Affiliations:** 1 Department of Social and Preventive Epidemiology, School of Public Health, University of Tokyo, Tokyo 113-0033, Japan; fujiwaraay@nibiohn.go.jp (A.F.); stssasak@m.u-tokyo.ac.jp (S.S.); 2 Nutrition Innovation Centre for Food and Health (NICHE), School of Biomedical Sciences, Ulster University, Coleraine BT52 1SA, UK; mbe.livingstone@ulster.ac.uk; 3 Department of Social and Preventive Epidemiology, Graduate School of Medicine, University of Tokyo, Tokyo 113-0033, Japan; nana-s@m.u-tokyo.ac.jp (N.S.); msugimoto@m.u-tokyo.ac.jp (M.S.); 4 Department of Nutritional Epidemiology and Shokuiku, National Institute of Biomedical Innovation, Health and Nutrition, Tokyo 162-8636, Japan; 5 Ikurien-naka, Ibaraki 311-0105, Japan; sizuko-masa@themis.ocn.ne.jp

**Keywords:** food combination, breakfast, lunch, dinner, snack, meal, diet quality, Japan, epidemiology

## Abstract

We examined food combinations in relation to the quality of the overall diet and individual meals using a newly developed food combination questionnaire (FCQ) in a nationwide sample of Japanese adults aged 19–80 years (*n* = 2233). The quality of the overall diet and of each meal was assessed by the Healthy Eating Index-2015 (HEI-2015) and Nutrient-Rich Food Index 9.3 (NRF9.3). For all main meals (breakfast, lunch, and dinner), the most commonly consumed food combinations consisted of ‘rice, total vegetables, and tea and coffee’. Consistently positive associations between these food combinations and diet quality were found for breakfast (Spearman r: ≥0.46). Positive rather weak associations between these food combinations and diet quality were also observed for lunch (Spearman r: ≤0.48). Conversely, the associations were inconsistent for dinner: inverse associations with HEI-2015 (Spearman r: ≤−0.35) and generally weak positive associations with NRF9.3 (Spearman r: ≥0.09). For snacks, the most commonly consumed food combinations consisted of ‘confectioneries and tea and coffee’, but these showed rather weak associations with diet quality. Similar results were obtained when associations with the quality of overall diet were investigated. The FCQ may be useful in capturing the complex nature of food combinations in Japanese adults.

## 1. Introduction

Efforts to overcome the limitations of evaluating single nutrients and foods in isolation have led to a gradual shift in nutrition research to the evaluation of dietary patterns [1,2]. Although the investigation of dietary patterns is generally performed in terms of the daily intake of individual foods or food groups [3,4,5,6], an increasing number of studies have focused on dietary intake at the level of each eating occasion (i.e., breakfast, lunch, dinner, and snack) or meal patterns [7,8,9]. Studying dietary patterns at this level rather than overall dietary patterns might be more relevant considering synergies and interactions during digestion and metabolism [10]. Recent studies also suggest that not only the amount and content of food intake but also the circadian timing of food intake need to be considered [11,12,13]. Understanding food combinations at meals could be useful for the development of public health nutrition policies and recommendations.

However, little is known about the combinations of foods consumed simultaneously during specific eating occasions [14,15,16,17,18,19,20,21,22,23], mainly because of a lack of practical assessment tools. There exist a near-infinite number of feasible food combinations, resulting in an unmanageable number of individual meals. Thus, a practical examination of meal patterns or food combinations requires the development of unique codes for meals [15], and then the development of inexpensive and practical assessment tools (e.g., questionnaires) [9,22,23]. Such a meal coding system will also be essential in the development of Internet-delivered personalized dietary analysis [24] as well as Internet-based self-administered dietary assessment tools [25,26].

We recently applied the ‘frequent item sets’ data-mining method [27] to data from 16 day weighed dietary records obtained from 242 Japanese adults to characterize combinations of foods served as a meal, and then developed a meal coding system [22]. Analysis of a total of 14,734 meals identified 80 generic meals. As one example, a meal code for breakfast was built on the combination of vegetables, tea and coffee, rice, pulses, fruit, and dairy products. Interrogation of these 80 meal codes by principal components analysis identified 11 interpretable meal patterns; examples include patterns characterized by three main meals consisting of the combination of rice and vegetables. Taken together, this meal coding system may be useful for developing a practical assessment tool for combinations of foods consumed simultaneously during specific eating occasions.

In the present study, we developed a self-administered dietary assessment questionnaire, namely a food combination questionnaire (FCQ), to characterize food combinations in each meal (breakfast, lunch, dinner, and snacks) on the basis of our meal coding system (food combination database). We then examined food combinations in relation to the quality of the overall diet and each meal in a nationwide sample of Japanese adults.

## 2. Materials and Methods

### 2.1. Study Procedure and Participants

This cross-sectional analysis was based on data obtained from a nationwide survey conducted between October and December 2018. The target population consisted of apparently healthy Japanese aged 18–80 years living in private households in Japan. Initially, 32 (of 47) prefectures, which account for >85% of the total population of Japan, were selected on the basis of geographical diversity and feasibility of the survey, particularly the recruitment of research dietitians as collaborators. After being recruited in person or by email, a total of 475 research dietitians agreed to support the study by collecting data. Although we did not provide them with any specific training for this study, all of them had experience in dietary data collection. They then conducted the recruitment of participants from local communities.

Based on feasibility and human and financial resources, we decided to include 474 individuals (237 for each sex) for each of six age groups: 18–29, 30–39, 40–49, 50–59, 60–69, and 70–80 years (*n* = 2844 in total). The non-random sampling procedure was performed to reflect the proportion of the overall Japanese population in each region: Hokkaido 4%, Tohoku 7%, Kanto I 28%, Kanto II 8%, Hokuriku 4%, Tokai 12%, Kinki I 13%, Kinki II 3%, Chugoku 6%, Shikoku 3%, Kita-kyushu 7%, and Minami-kyushu 5% [28]. Inclusion criteria consisted of willingness to participate and community-dwelling (free-living) individuals. Exclusion criteria were dietitians, individuals living together with a dietitian, those working together with a research dietitian, those who had experienced dietary counseling from a doctor or dietitian, those taking insulin treatment for diabetes, those receiving dialysis treatment, and pregnant or lactating women. Participation of only one person per household was permitted. Consequently, a total of 2248 individuals participated in this study (response rate 79%).

Participants were asked to answer two questionnaires sequentially on dietary habits, namely the FCQ and a brief diet history questionnaire (BDHQ). Responses to both questionnaires were thoroughly checked by research dietitians and then by the first author (K.M.) at the study center. If any missing or erroneous responses were given, the participant was asked to complete the questions again in person or by telephone. For analysis, we excluded participants who did not answer both questionnaires (*n* = 3) and those aged outside the 18–80 year age range (*n* = 12), leaving 2233 participants aged 19–80 years. A flow diagram of participants included in the present analysis is shown in Figure S1.

The study was conducted according to the guidelines laid down in the Declaration of Helsinki and all procedures involving human subjects were approved by the Ethics Committee of the University of Tokyo Faculty of Medicine (number 12031). Written informed consent was obtained from each participant, and from a parent or guardian for participants aged < 20 years.

### 2.2. BDHQ

#### 2.2.1. General Description

Details of the BDHQ’s structure and calculation method of dietary intake have been published elsewhere [29,30]. In brief, the BDHQ is a four page self-administered questionnaire on dietary habits during the preceding month which generally takes 15 min to answer. It consists of structured questions asking about the consumption frequency of selected foods commonly consumed in Japan, as well as general dietary behavior and usual cooking methods. Estimates of daily intake of foods (58 items in total), energy, and selected nutrients were calculated using an ad hoc computer algorithm for the BDHQ, which incorporates the sex-specific portion size, determined mainly based on recipe books for Japanese dishes [29], and nutrient composition of each food item derived from the Standard Tables of Food Composition in Japan [31]. It should be noted that only overall dietary intake was calculated from the BDHQ.

The validity of the BDHQ was examined in 92 women and 92 men using a 16 day weighed dietary record as reference [29,30]. In brief, the median of Spearman correlation coefficients for food groups was 0.44 (range 0.14–0.82) in women and 0.48 (range 0.22–0.83) in men [29], while the median of Pearson correlation coefficients for nutrients was 0.54 (range 0.27–0.84) in women and 0.56 (range 0.19–0.81) in men [30].

#### 2.2.2. Calculation of Diet Quality Scores

In this study, we used the Healthy Eating Index 2015 (HEI-2015) [32,33,34] and Nutrient-Rich Food Index 9.3 (NRF9.3) [35,36,37,38] as measures of diet quality. The HEI-2015 is a 100 point scale to assess compliance with the 2015–2020 Dietary Guidelines for Americans [39], with a higher score indicating a better quality of overall diet. The HEI-2015 consists of nine adequacy components (total fruits, whole fruits, total vegetables, greens and beans, whole grains, dairy, total protein foods, seafood and plant proteins, and fatty acids as the ratio of the sum of polyunsaturated and monounsaturated fatty acids to saturated fatty acids) and four moderation components (refined grains, sodium, added sugars, and saturated fats). As described elsewhere [40], we calculated the HEI-2015 component and total scores based on energy-adjusted values of overall dietary intake, namely amount per 1000 kcal of energy or percentage of energy, except for fatty acids.

The NRF9.3 is a composite measure of the nutrient density of the total diet, calculated as the sum of the percentage of reference daily values (RDVs) for nine qualifying nutrients, namely protein, dietary fiber, vitamin A, vitamin C, vitamin D, calcium, iron, potassium, and magnesium, minus the sum of the percentage of RDVs for three disqualifying nutrients, namely added sugars, saturated fats, and sodium. RDVs were determined for sex and age categories, based on the Dietary Reference Intakes (DRIs) for Japanese, 2015 [41], except for added sugars, for which the conditional recommendation advocated by the World Health Organization (i.e., upper limit of 5% of energy) [42] was used because of the lack of a recommended value for added sugars in Japan, as well as their low intake levels [43]. As described elsewhere [40], we calculated the NRF9.3 component and total scores based on the overall daily intake of each nutrient for each participant, which was adjusted for energy intake by the density method and then normalized for the sex- and age-specific Estimated Energy Requirement for a moderate level of physical activity (from DRIs) and expressed as a percentage of the RDV. Higher NRF9.3 scores indicated a better quality of the overall diet.

The validity of the BDHQ in terms of HEI-2015 and NRF9.3 has been previously investigated against a 16 day weighed dietary record as reference [40]. Briefly, the Pearson correlation coefficient for the former was 0.52 in women (*n* = 121) and 0.43 in men (*n* = 121), while that for the latter was 0.61 in women and 0.37 in men.

### 2.3. FCQ

#### 2.3.1. Food Combination Database

The basis of the FCQ is a food combination database (meal coding system) which was recently developed by our research group using four day weighed dietary record data collected in each season over a one year period (16 days in total) from 242 Japanese adults aged 31–81 years; the food combination database used in this study is shown in Table S1. A detailed description of the development of the database has been published elsewhere [22], and Figure S2 shows a flow diagram of the development process. Briefly, for all meal types, namely breakfast (*n* = 3788), lunch (*n* = 3823), dinner (*n* = 3856), and snacks (*n* = 3267), we categorized the most commonly consumed combinations of 17 selected food groups according to the ‘frequent item sets’ data-mining method [27]. An example of this procedure is shown in Figure S3. We estimated the nutrient content of each coded generic meal using the aggregation of the nutrient composition of individual meals assigned to that code. For each meal for each individual, we calculated the total amount (g), weight of each food group, and content of each nutrient using the Standard Tables of Food Composition in Japan [44]. We then calculated mean values of these variables based on all meals classified for each meal code. A compilation of these mean values thus comprised the food combination database. In this study, all the food combinations (meal codes) except for “all other combinations” for each meal were used (*n* = 76).

#### 2.3.2. Development of FCQ

**Figure 1:**
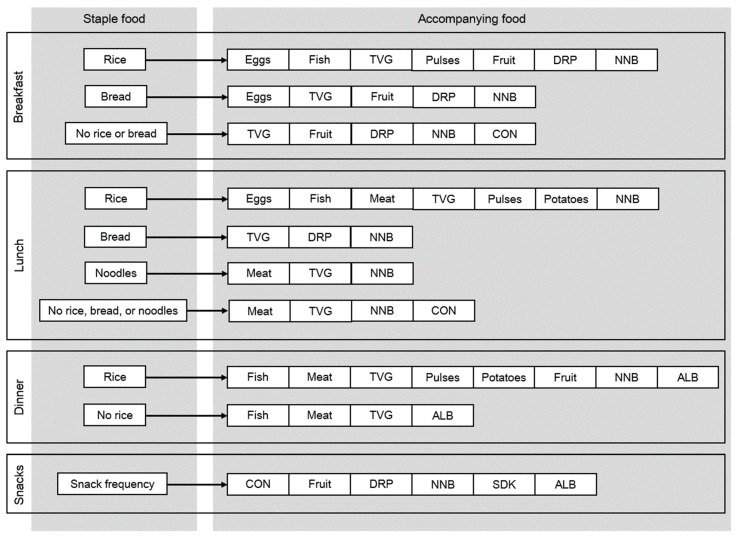
Structure of the food combination questionnaire (FCQ). In the FCQ, consumption frequency of each staple food in each meal type was enquired about in terms of the number of days with consumption per week during the preceding month; for snacks, consumption frequency was similarly enquired about without specifying any staple foods. For accompanying foods for each staple food, relative consumption frequency was enquired about, namely how often the food was consumed with the staple food, with the possible answers of ‘always’, ‘sometimes’, and ‘never’. The food group ‘fish’ includes shellfish; the food group ‘pulses’ includes nuts. ALB, alcoholic beverages; CON, confectioneries; DRP, dairy products; NNB, tea and coffee (i.e., nonalcoholic and noncaloric beverages); SDK, soft drinks; TVG, total vegetables.

Our priority in developing the FCQ was to collect information which is sufficient to distinguish food combinations using as few questions as possible. Careful scrutiny of the food combination database showed that there was no meal code in which staple foods for Japanese (i.e., rice, bread, and noodles) appeared in combination, whereas many meal codes included at least one of these staple foods. We thus considered that a format consisting of questions on staple foods followed by questions on accompanying foods is the best for FCQ. Figure 1 shows the structure of the FCQ. Based on the food combination database (meal codes), staple foods in the FCQ were defined as follows: rice and bread for breakfast; rice, bread, and noodles for lunch; rice for dinner; and no staple food for snacks. For each staple food for each meal type, accompanying foods were then defined as food groups which had contributed to the determination of meal codes in the development of food combination database [22], so that we could collect minimum information for distinguishing food combinations.

In the FCQ, we asked about consumption frequency as the number of days the food was consumed per week for each staple food for each meal type; for snacks, consumption frequency was asked about in a similar way without specifying any staple foods. The reference time period was defined as the preceding month, which was in accordance with the BDHQ. For accompanying foods, we asked about relative consumption frequency, namely how often the food was consumed with the staple food, with possible answers of ‘always’, ‘sometimes’, and ‘never’. The developed FCQ was a four page, self-administered questionnaire, which generally took 5 min to answer according to a pretest conducted among 19 individuals.

#### 2.3.3. Development of an Algorithm for Determining Food Combinations

We developed an ad hoc computer algorithm for determining the food combinations consumed by each participant based on the information collected from the FCQ. An example of the calculation of the daily consumption frequency of food combinations is described in Figure S4. First, based on the possible answers for accompanying foods, namely ‘always’, ‘sometimes’, and ‘never’, in the FCQ, each of the food combinations (meal codes) was characterized in the same manner. For this, foods labeled as ‘always’ were those always included in the meal code, foods labeled as ‘never’ were those not included (always excluded) in the meal code, and foods labeled as ‘sometimes’ were all other foods. All the answers were then coded based on the coding rule. A coefficient value was then calculated for each meal code for each staple food for each meal. If the coefficient value was negative, a value of zero was assigned. Finally, the consumption frequency of each meal code was calculated based on the consumption frequency of the staple food weighted by the coefficient value as a percentage of the sum of coefficient values with the same staple food in each meal.

#### 2.3.4. Calculation of Dietary Intakes

Generally speaking, estimates of daily intakes of food groups, energy, and nutrients from each meal code were calculated as the daily consumption frequency of each food combination (meal code) multiplied by the composition of each meal code. However, a different calculation procedure was used for accompanying foods, as well as the nutrients and energy derived from these foods, for each staple food for each meal. As described in Figure S5, the daily consumption frequency of accompanying foods was calculated as the daily consumption frequency of the corresponding staple foods multiplied by a factor determined based on the answer for relative consumption frequency. Estimates of daily intakes of accompanying foods were then calculated as the consumption frequency multiplied by the average composition, which was weighted by the number of appearances in the original meal composition database [22], of all meal codes included in the respective staple food (for example, meal codes 1101–1108, 1301, 1302, and 1401 for rice for breakfast). Daily intakes as well as intakes from each meal were calculated by summing all of the estimates calculated as described above.

#### 2.3.5. Calculation of Diet Quality Scores

Before development of the food combination database, we merged the original database, which consisted of individual food items compiled in the Standard Tables of Food Composition in Japan [44], with a cup and ounce equivalent database needed to estimate the HEI-2015 [40]. Thus, all of the estimates needed to calculate the HEI-2015 were obtained in the same way as the calculation of intakes of food groups, energy, and nutrients, described in the previous section. For the overall diet as well as for each meal, both the HEI-2015 and NRF9.3 were calculated based on the information derived from the FCQ, using the same procedure used in the BDHQ.

We compared overall diet quality scores and overall dietary intakes assessed by the FCQ and those assessed by the BDHQ. As shown in Table S2, the Spearman correlation coefficients for total scores of HEI-2015 and NRF9.3 were 0.49 and 0.48, respectively, while the median value of Spearman correlation coefficients for 19 food groups was 0.42 (range 0.07 to 0.82). These results suggest that the FCQ potentially has sufficient ability to estimate intakes, for the overall diet at least.

### 2.4. Statistical Analysis

All statistical analyses were performed using SAS statistical software (version 9.4, SAS Institute Inc., Cary, NC, USA). Data are presented as means ± standard deviations for diet quality scores and as medians and 25th and 75th percentiles for food group intakes and food combinations for each meal. Spearman correlation coefficients were calculated among diet quality scores. Associations between food group intakes in each meal and diet quality scores for each meal were examined using the Spearman correlation coefficients. Associations between food combinations in each meal and diet quality scores for each meal were also examined using the Spearman correlation coefficients. Finally, associations between food combinations in each meal and overall diet quality scores were examined using the Spearman correlation coefficients.

## 3. Results

The present analysis included 2233 Japanese adults (1070 men and 1163 women aged 19–80 years) with a mean age of 50 years (Table 1). The mean HEI-2015 for overall diet was 53.3 (standard deviation 2.7) while the mean NRF9.3 for overall diet was 709 (standard deviation 56).

### 3.1. Quality of Breakfast, Lunch, Dinner, and Snacks

In this population, dinner was, on average, the top contributor to total energy intake, followed, in order, by lunch, breakfast, and snacks (Table 2). Diet quality as assessed by the HEI-2015 and NRF9.3 was also highest for dinner, followed by lunch, breakfast, and snacks. As theoretically expected, the quality of each meal was positively correlated with that of the overall diet (Spearman r: 0.29–0.71 for HEI-2015 and 0.33–0.71 for NRF9.3) (Table S3). Nevertheless, the correlation among meals was relatively weak (Spearman r: 0.11–0.21 for HEI-2015 and 0.16–0.38 for NRF9.3). The correlation between HEI-2015 and NRF9.3 was considerably high for total diet, breakfast, lunch, and snacks, but not for dinner (Spearman r: 0.67, 0.82, 0.75, 0.77, and 0.17, respectively).

### 3.2. Food Group Intake in Breakfast, Lunch, Dinner, and Snacks

For breakfast and lunch (Table 3) as well as snacks (Table S4), the median of tea and coffee consumption was the largest among the 20 food groups considered in this study. However, the next most commonly consumed food groups (i.e., >50 g/1000 kcal) differed considerably, namely rice, dairy products, seasonings, total vegetables, and bread for breakfast; rice, seasonings, total vegetables, and noodles for lunch; and confectioneries, dairy products, soft drinks, and fruit for snacks. For dinner (Table 3), total vegetables was the most commonly consumed food group, followed by rice, tea and coffee, and seasonings.

The associations between intakes of these major food groups and diet quality also differed considerably for each meal. In breakfast, total vegetables and rice as well as seasonings were positively associated with diet quality, while bread showed an inverse association (with less clear inverse associations for tea and coffee or dairy products). For lunch, only total vegetables showed a positive association with diet quality. For dinner, there was an inverse association of rice with HEI-2015 (but not NRF9.3) and of seasonings with NRF9.3 (but not HEI-2015), while total vegetables showed positive associations with both HEI-2015 and NRF9.3. For snacks, there were positive associations for tea and coffee and fruits as well as dairy products, while confectioneries and soft drinks showed inverse associations. 

### 3.3. Food Combinations in Breakfast, Lunch, Dinner, and Snacks

For breakfast, the most commonly consumed food combinations consisted of ‘rice, total vegetables, and tea and coffee’ (meal codes 1101–1108) which, in total, contributed to 7.0% of total energy intake (Table 4). These meal codes, as well as those consisting of rice accompanied by total vegetables, tea and coffee, or both (meal codes 1301, 1302, and 1401), were consistently positively associated with the quality of the breakfast. On the other hand, the meal codes consisting of bread, which were frequently accompanied by total vegetables, dairy products, and tea and coffee (meal codes 1109–1111, 1201–1204, and 1501), were generally inversely associated with the quality of the breakfast.For lunch (Table S5) and dinner (Table S6), the most commonly consumed food combinations again consisted of ‘rice, total vegetables, and tea and coffee’ (meal codes 2101–2110 for lunch and 3101–3111 for dinner), which, in total, contributed to 14.6% and 18.5% of total energy intake, respectively. Nevertheless, the associations between these meal codes and diet quality were weak in the case of lunch, compared with the case of breakfast. For dinner, the associations were inconsistent, namely the meal codes showing inverse associations with HEI-2015 but generally weak positive associations with NRF9.3. For snacks, the most commonly consumed food combinations consisted of “confectioneries and tea and coffee” (meal codes 4101–4103), but these showed rather weak associations with diet quality (Table S7). On the other hand, the combinations of ‘dairy products and tea and coffee’ and ‘fruit and tea and coffee’ (meal codes 4201 and 4301, respectively) showed positive associations with diet quality. These associations, observed in each meal, were generally consistent with those between food combinations and the quality of the overall diet (Table 5).

## 4. Discussion

Using the FCQ based on a meal coding system, we found that the most commonly consumed food combinations consisted of ‘rice, total vegetables, and tea and coffee’ for all main meals in Japanese adults. For breakfast, these food combinations were consistently positively associated with diet quality, as assessed by the HEI-2015 and NRF9.3. The associations were similar in direction for lunch but rather weak. For dinner, however, the associations were inconsistent: there were inverse associations with HEI-2015 but weak positive associations with NRF9.3. For snacks, the most commonly consumed food combinations consisted of ‘confectioneries and tea and coffee’, but these showed rather weak associations with diet quality. Thus, associations between the common food combinations and diet quality differed among meals. These diverse associations were confirmed when associations with the quality of the overall diet, rather than each meal, were investigated. To our knowledge, this is the first epidemiologic study to comprehensively investigate food combinations in each meal in relation to diet quality.

Japanese dietary habits have long attracted interest from other countries, primarily because of their possible contribution to a low prevalence of coronary artery disease and long life expectancy [45,46]. According to a secondary analysis of one day dietary record data obtained from 15,618 Japanese adults in the 2012 National Health and Nutrition Survey, breakfast was characterized by a high (>30 g/d) intake of tea and coffee, rice, vegetables, dairy products, and fruit; lunch by a high intake of tea and coffee, rice, vegetables, and noodles; dinner by a high intake of vegetables, rice, alcoholic beverages, tea and coffee, fish, meat, seasonings, potatoes, and pulses; and snacks by a high intake of tea and coffee and fruit [47]. In the same study, breakfast, lunch, dinner, and snacks on average contributed to 23%, 30%, 40%, and 8% of total energy intake, respectively [47]. Additionally, the most frequently identified food combination for all three main meals was ‘rice and vegetables’, whereas ‘confectioneries and tea and coffee’ was the most prevalent combination for snacks [23]. With regard to the HEI-2015 and NRF9.3, mean values derived from a 16 day weighed dietary record were 55.4 and 704 in 121 women and 54.3 and 728 in 121 men, respectively [40]. These are generally consistent with the present findings, suggesting the utility of the FCQ and the meal coding system for characterizing dietary intake and meal patterns in Japanese as well as the robustness of the present findings.

In this study, the associations of diet quality were rather weak among meals, irrespective of diet quality measure. The reason is unknown, but may be due to the low consistency of food intake among meals, as well as the somewhat independent nature of each meal, at least in terms of quality rather than quantity. This is not inconsistent with a within-person comparison of daily dietary intake with and without breakfast, in which there were no differences in the quality of foods selected, as reflected in energy density and energy-adjusted intakes of energy-providing nutrients and dietary fiber [48]. The present findings in turn suggest the importance of accumulating evidence at the meal level to develop effective meal-level dietary guidelines.

Interestingly, we found that while the common food combinations were ‘rice, total vegetables, and tea and coffee’ in all main meals, the associations between these food combinations and diet quality were rather different among these meals. This may be due to differences in the associations between food group intake and diet quality. For example, the association between rice and diet quality was positive for breakfast, almost null for lunch, and inverse (for HEI-2015) or almost null (for NRF9.3) for dinner. Additionally, the positive association between total vegetables and diet quality was quite strong for breakfast and lunch but moderate for dinner. Another reason may be that, while there were multiple options for staple foods in breakfast (rice and bread) and lunch (rice, bread, and noodles), only rice was available as a staple food in dinner. Because of this, while the quality of the common food combinations mainly comprising rice was generally assessed by comparison with the quality of food combinations comprising other staple foods (which were generally inversely associated with diet quality) in breakfast and lunch, the quality of the common food combinations was assessed within the same staple food, namely rice, in dinner, which may cause an inverse association between rice and HEI-2015. In any case, these observations highlight the complex nature of food combinations, which, in turn, suggests the importance of this kind of research.

In this study, the mean quality of breakfast was lower than that of lunch and dinner. This may be mainly due to high intakes of rice and bread—major sources of energy—as well as dairy products—a major source of saturated fatty acids—in breakfast, making the energy-adjusted values of other important foods and nutrients decrease. The present finding is consistent with recent analyses of national dietary survey datasets, in which the nutrient density of breakfast per se was, on average, low in Japan [49] compared with Western countries [50]. On the other hand, the variation (standard deviation) in diet quality was higher in breakfast than in lunch and dinner. Because of this, food combinations in breakfast were more strongly associated with the quality of the overall diet compared with those in lunch and dinner, as well as those in snacks, whose contribution to overall diet was rather small. Consequently, improvement in the quality of breakfast may be not only the most important strategy for improving overall diet quality in Japanese adults, but also the most feasible. On the other hand, a considerably different picture was observed in German adults, in whom dinner was the greatest contributor to the formation of the four overall dietary patterns identified [18]. The reason for this discrepancy is unknown but may include differences in the study populations, dietary intakes and habits, and dietary assessment methods. Nevertheless, both studies clearly showed that overall dietary intakes or patterns originate, to some extent, at the meal level, which could, in turn, lead to a better understanding of how dietary patterns, meal patterns, and food combinations arise. This kind of basic information should be accumulated from various countries.

Recently, chrono-nutrition has been emphasized in the field of nutritional epidemiology because of the potential importance of the timing, in addition to composition, of dietary intake [11,12,13]. For example, in a British cohort, increasing energy intake from carbohydrates at the expense of a similar amount of energy from fat at breakfast and at mid-morning at the age of 43 years was associated with decreased prevalence of metabolic syndrome 10 years later [51]. Another six year prospective cohort study in Italy has shown that a higher intake of energy at dinner was associated with higher incidence of obesity, metabolic syndrome, and non-alcoholic fatty liver disease [52]. Further, a traditional wheat-based breakfast identified by factor analysis was associated with a decreased risk of hyperglycemia in a six year prospective cohort study in Chinese adults, whereas a rice-based traditional lunch and dinner was associated with an increased risk [53]. Because the FCQ can provide information on dietary intake at each meal, it might be a promising tool for this kind of research, as well as in investigating the associations between food combinations and various health outcomes in large-scale epidemiologic studies.

The strengths of the present study include its use of the FCQ and a meal coding system, which were empirically developed based on detailed information on actual food combinations over a one year period with a large number of recording days (16 days) in 242 Japanese [22]. However, there are also several limitations. First, although sampling was conducted to reflect regional differences in population proportion, the present population is not a nationally representative sample of general Japanese, but rather volunteers. In particular, our participants may be biased toward greater health consciousness. Further research in a more representative sample is needed.

Second, the nature and extent of the measurement error of self-reported information on food combinations obtained by the FCQ are largely unknown. The present results should therefore be interpreted with caution in this respect. Nevertheless, comparison of the FCQ with the BDHQ, a widely used and well-validated dietary assessment questionnaire [29,30], suggests that the FCQ may have sufficient ability to estimate overall dietary intakes and diet quality scores. Additionally, all the dietary variables used in this study were energy-adjusted to minimize the influence of measurement error in self-reported dietary intake [54]. In any case, more rigorous validation assessment of the FCQ is needed, particularly for characterizing dietary intake and food combinations in each meal, notwithstanding the lack of objective markers of food combinations [48] and the fact that even more extensive dietary assessment methods, such as dietary record and 24 hour recall, not only rely on self-reporting but are themselves also subject to both random and systematic measurement errors [54,55,56].

Third, because the survey was conducted within a three month period (from October to December 2018) and the FCQ assessed dietary habits during the preceding month, any seasonal variation in food combinations was not considered. Given that several previous studies have observed seasonal differences in intakes of at least some nutrients and food groups in Japanese adults [57,58,59], this might have produced some bias in assessing average food combinations over the year. Additionally, because of the design of the study, we do not know how much intra-person variability in food combinations and meal intake there is; there would be some compensation within individuals across meals and across days. Further studies in these respects are needed.

Finally, we used the HEI-2015 and NRF9.3 in this Japanese study, even though both scores were primarily developed to assess the overall diet quality of Americans. Thus, these diet quality measures are not optimal for assessing the overall quality of Japanese diet, but rather the best available [40]. The use of other diet quality scores, such as the Dietary Inflammatory Index [1], which is not culture bound, would be of interest in future studies. Nevertheless, in our recent systematic review of Japanese studies which obtained dietary patterns using principal component analysis, we found that those food groups which contributed to dietary patterns termed healthy (fruits, vegetables, potatoes, mushrooms, seaweeds, and pulses) are at least partly similar to those often observed in Western countries (fruits, vegetables including mushrooms, poultry, fish, low-fat dairy, legumes, and whole grains) [60]. It should also be stressed that our recent analysis supports the efficacy of these measures in assessing the overall diet quality of Japanese: a higher total score in the HEI-2015 and NRF9.3 was associated with favorable patterns of overall diet, including higher intakes of dietary fiber and key vitamins and minerals and lower intakes of saturated fats [40].

## 5. Conclusions

In this study, the most commonly consumed food combinations consisted of ‘rice, total vegetables, and tea and coffee’ for all main meals, including breakfast, lunch, and dinner, but associations between these common food combinations and diet quality differed among meals. The FCQ and meal coding system used here may be useful in capturing the complex nature of food combinations in Japanese adults. Further methodological studies on whether similar methods for characterizing food combinations within meals may be applicable to other populations would also be of interest.

## Figures and Tables

**Table 1:** Basic characteristics of the study population ^a^.

Variable	Total (*n* = 2233)	Men (*n* = 1070)	Women (*n* = 1163)
Age (years)	50.1 ± 17.3	50.3 ± 17.2	50.0 ± 17.5
Body height (cm) ^b^	162.6 ± 8.9	169.4 ± 6.3	156.3 ± 5.9
Body weight (kg) ^b^	60.9 ± 12.1	68.0 ± 10.9	54.4 ± 9.0
Body mass index (kg/m^2^) ^c^	22.9 ± 3.5	23.7 ± 3.3	22.3 ± 3.5

^a^ Values are means ± standard deviations. ^b^ Based on self-report. ^c^ Calculated using self-reported body height and weight.

**Table 2:** Energy intake from each meal and diet quality score for each meal in 2233 Japanese adults aged 19–80 years ^a^.

Variable	Maximum Score	Breakfast	Lunch	Dinner	Snacks
Energy (kcal/day)	---	382 ± 111	551 ± 100	621 ± 69	101 ± 54
Percentage of total energy	---	22.8 ± 5.8	33.2 ± 5.3	37.9 ± 5.4	6.1 ± 3.1
HEI-2015 ^b^	100	46.8 ± 7.7	52.2 ± 2.9	55.3 ± 4.2	46.4 ± 6.6
Total fruits	5	1.7 ± 1.4	1.9 ± 0.5	1.1 ± 0.6	3.0 ± 1.9
Whole fruits	5	2.6 ± 2.0	3.6 ± 0.7	2.0 ± 1.1	3.3 ± 2.3
Total vegetables	5	3.5 ± 1.7	4.0 ± 1.2	4.9 ± 0.6	2.1 ± 0.7
Greens and beans	5	2.9 ± 1.6	2.6 ± 1.0	4.4 ± 0.9	2.1 ± 0.5
Whole grains	10	0.5 ± 0.3	1.4 ± 0.6	0.7 ± 0.1	0.3 ± 0.2
Dairy	10	4.3 ± 3.2	1.6 ± 1.5	1.0 ± 0.2	3.3 ± 2.5
Total protein foods	5	3.6 ± 1.0	3.7 ± 0.7	5.0 ± 0.2	2.7 ± 0.8
Seafood and plant proteins	5	4.1 ± 1.2	4.7 ± 0.9	5.0 ± 0.3	4.8 ± 1.0
Fatty acids	10	4.9 ± 3.2	8.7 ± 1.9	9.8 ± 0.5	1.7 ± 2.3
Refined grains	10	0.4 ± 1.7	0.0 ± 0.7	1.6 ± 2.8	3.9 ± 2.5
Sodium	10	1.1 ± 2.1	0.1 ± 0.8	0.0 ± 0.3	9.9 ± 0.4
Added sugars	10	9.3 ± 2.1	10.0 ± 0.2	10.0 ± 0.0	1.1 ± 2.1
Saturated fats	10	8.0 ± 2.7	9.8 ± 0.8	9.8 ± 0.6	8.1 ± 1.7
NRF9.3 ^c^	900	610 ± 277	623 ± 78	733 ± 61	281 ± 107
Protein	100	99 ± 10	100 ± 4	100 ± 3	91 ± 20
Dietary fiber	100	78 ± 18	74 ± 13	86 ± 15	68 ± 18
Vitamin A	100	59 ± 22	64 ± 16	96 ± 9	53 ± 14
Vitamin C	100	81 ± 25	87 ± 15	95 ± 12	92 ± 22
Vitamin D	100	81 ± 24	81 ± 22	98 ± 10	46 ± 14
Calcium	100	87 ± 20	66 ± 15	75 ± 13	83 ± 23
Iron	100	88 ± 18	90 ± 16	96 ± 9	89 ± 22
Potassium	100	89 ± 15	78 ± 11	96 ± 8	94 ± 21
Magnesium	100	90 ± 13	81 ± 8	98 ± 6	91 ± 20
Added sugars	--- ^d^	51 ± 200	3 ± 11	1 ± 4	393 ± 100
Saturated fats	--- ^d^	30 ± 36	4 ± 13	6 ± 11	33 ± 22
Sodium	--- ^d^	61 ± 32	90 ± 29	101 ± 26	0 ± 2

HEI-2015, Healthy Eating Index-2015; NRF9.3, Nutrient-Rich Food Index 9.3. ^a^ Values are means ± standard deviations. A higher score indicates a higher diet quality, except for added sugars, saturated fats, and sodium components in NRF9.3, for which a higher score indicates an unfavorable dietary intake (i.e., higher intakes of added sugars, saturated fats, and sodium). ^b^ Calculated as the sum of all component scores. ^c^ Calculated as the sum of scores for nine nutrients to encourage (i.e., protein, dietary fiber, vitamins A, C, and D, calcium, iron, potassium, and magnesium) minus the sum of scores for three nutrients to limit (i.e., added sugar, saturated fats, and sodium). ^d^ A maximum score is infinite depending on the intake level.

**Table 3:** Food group intake in breakfast, lunch, or dinner in relation to diet quality scores for each meal in 2233 Japanese adults aged 19–80 years ^a^.

			Breakfast					Lunch					Dinner		
Food	Intake (g of wet weight per 1000 kcal of energy from breakfast)			r with HEI-	r with	Intake (g of wet weight per 1000 kcal of energy from lunch)			r with HEI-	r with	Intake (g of wet weight per 1000 kcal of energy from dinner)			r with HEI-	r with
Group	Median	P25	P75	2015	NRF9.3	Median	P25	P75	2015	NRF9.3	Median	P25	P75	2015	NRF9.3
Rice	180.7	3.1	284.8	0.52	0.56	231.8	182.3	270.9	0.06	0.16	215.7	158.2	245.4	−0.72	0.11
Bread	53.1	3.3	141.2	−0.59	−0.37	3.2	1.7	24.4	−0.10	−0.13	2.2	1.2	4.3	0.63	−0.37
Noodles	3.4	1.3	4.4	0.36	0.11	74.0	64.4	122.8	−0.05	−0.34	18.5	15.6	33.6	0.17	−0.22
Pulses ^b^	22.6	9.9	30.1	0.69	0.57	15.2	14.0	16.7	0.19	0.06	31.3	25.9	43.4	0.46	0.03
Total vegetables	87.6	44.6	139.7	0.84	0.84	88.9	73.9	145.1	0.76	0.75	221.1	131.7	241.3	0.35	0.56
Fruit	34.6	0	51.9	0.41	0.25	41.8	38.0	46.9	−0.12	−0.19	23.5	19.7	33.3	0.85	−0.07
Fish ^c^	10.6	4.8	17.8	0.57	0.59	25.0	20.8	27.7	0.32	0.23	43.6	40.7	46.3	0.03	−0.11
Meat	16.3	14.7	18.1	−0.58	−0.65	21.4	19.1	23.7	0.10	0.24	37.2	34.3	46.2	0.30	−0.30
Dairy products	120.9	62.8	190.8	−0.17	−0.10	19.7	17.5	56.0	−0.17	−0.13	23.7	21.7	26.9	0.49	−0.44
Alcoholic beverages	0.5	0.2	0.6	0.72	0.65	13.4	11.1	15.4	0.33	0.33	29.0	0	140.7	0.31	0.04
Tea and coffee ^d^	404.5	363.2	457.8	−0.17	−0.29	301.5	239.1	329.3	0.05	−0.06	209.5	138.5	230.8	−0.22	−0.07
Seasonings	108.0	44.3	152.8	0.59	0.57	99.5	90.9	108.5	0.02	−0.24	115.5	109.0	120.6	0.12	−0.44

HEI-2015, Healthy Eating Index-2015; NRF9.3, Nutrient-Rich Food Index 9.3; P25, 25th percentile; P75, 75th percentile. ^a^ Spearman correlation coefficients between intakes of food groups and HEI-2015 and NRF9.3 were calculated according to each meal. For both HEI-2015 and NRF9.3, a higher score indicates a higher diet quality. Only data on the food groups whose median value was >25 g/1000 kcal at least for one meal category are shown. ^b^ Including nuts. ^c^ Including shellfish. ^d^ Consisting of nonalcoholic and noncaloric beverages.

**Table 4:** Food combinations (meal codes) in breakfast in relation to diet quality scores for breakfast in 2233 Japanese adults aged 19–80 years ^a^.

	Food Group Included		Intake (% of total energy)				
Meal Code	Staple Food	Accompanying Food ^b^	Median	P25	P75	r with HEI-2015	r with NRF9.3
1101	Rice	Total vegetables, tea and coffee, pulses, fruit, dairy products	0.86	0	2.25	0.77	0.82
1102	Rice	Total vegetables, tea and coffee, pulses, fruit	0.88	0	2.01	0.75	0.75
1103	Rice	Total vegetables, tea and coffee, pulses, eggs	1.09	0	2.35	0.63	0.71
1104	Rice	Total vegetables, tea and coffee, pulses, fish	0.73	0	1.78	0.64	0.72
1105	Rice	Total vegetables, tea and coffee, pulses	0.87	0	1.67	0.52	0.58
1106	Rice	Total vegetables, tea and coffee, eggs	1.15	0	2.12	0.59	0.67
1107	Rice	Total vegetables, tea and coffee, fish	0.72	0	1.55	0.59	0.67
1108	Rice	Total vegetables, tea and coffee	0.81	0	1.53	0.46	0.50
1109	Bread	Total vegetables, tea and coffee, dairy products, eggs	0.62	0	2.07	−0.18	0.03
1110	Bread	Total vegetables, tea and coffee, dairy products	0.58	0	1.90	−0.36	−0.14
1111	Bread	Total vegetables, tea and coffee	0.50	0	1.46	−0.34	−0.16
1112	No staple food	Total vegetables, tea and coffee	0	0	0	−0.09	−0.33
1201	Bread	Dairy products, tea and coffee, fruit	0.53	0	1.79	−0.35	−0.16
1202	Bread	Dairy products, tea and coffee	0.58	0	1.72	−0.62	−0.38
1203	Bread	Dairy products, total vegetables	0.33	0	1.05	−0.18	0.00
1204	Bread	Dairy products	0.29	0	0.98	−0.43	−0.25
1301	Rice	Total vegetables, dairy products	0.81	0	1.72	0.63	0.71
1302	Rice	Total vegetables	0.68	0	1.32	0.55	0.56
1401	Rice	Tea and coffee	0.80	0	1.29	0.46	0.48
1501	Bread	Tea and coffee	0.58	0	1.75	−0.51	−034
1601	No staple food	Dairy products, tea and coffee	0	0	0	−0.15	−0.41
1701	No staple food	Tea and coffee	0	0	0.05	−0.23	−0.48

HEI-2015, Healthy Eating Index-2015; NRF9.3, Nutrient-Rich Food Index 9.3; P25, 25th percentile; P75, 75th percentile. ^a^ Spearman correlation coefficients between intakes of meal codes in breakfast (as assessed by percentage of total energy intake) and the quality of breakfast as assessed by the HEI-2015 and NRF9.3 were calculated. For both HEI-2015 and NRF9.3, a higher score indicates a higher diet quality. ^b^ ‘Tea and coffee’ consisting of nonalcoholic and noncaloric beverages; ‘pulses’ including nuts; ‘fish’ including shellfish.

**Table 5:** Spearman correlation coefficients of food combinations in each meal (meal codes) expressed as percentage of total energy intake with the quality of the overall diet as assessed by the Healthy Eating Index-2015 (HEI-2015) and Nutrient-Rich Food Index 9.3 (NRF9.3) of overall diet among 2233 Japanese adults aged 19–80 years ^a^.

Meal		Food Group Included		r with	r with
Code	Meal Type	Staple Food	Accompanying Food	HEI-2015	NRF9.3
1101	Breakfast	Rice	Total vegetables, tea and coffee, pulses, fruit, dairy products	0.46	0.44
1102	Breakfast	Rice	Total vegetables, tea and coffee, pulses, fruit	0.44	0.38
1103	Breakfast	Rice	Total vegetables, tea and coffee, pulses, eggs	0.31	0.35
1104	Breakfast	Rice	Total vegetables, tea and coffee, pulses, fish	0.32	0.34
1105	Breakfast	Rice	Total vegetables, tea and coffee, pulses	0.22	0.26
1106	Breakfast	Rice	Total vegetables, tea and coffee, eggs	0.30	0.31
1107	Breakfast	Rice	Total vegetables, tea and coffee, fish	0.31	0.30
1112	Breakfast	No staple food	Total vegetables, tea and coffee	−0.02	−0.21
1201	Breakfast	Bread	Dairy products, tea and coffee, fruit	−0.22	−0.12
1202	Breakfast	Bread	Dairy products, tea and coffee	−0.50	−0.29
1204	Breakfast	Bread	Dairy products	−0.37	−0.22
1301	Breakfast	Rice	Total vegetables, dairy products	0.33	0.34
1302	Breakfast	Rice	Total vegetables	0.25	0.23
1501	Breakfast	Bread	Tea and coffee	−0.41	−0.30
1601	Breakfast	No staple food	Dairy products, tea and coffee	−0.05	−0.24
1701	Breakfast	No staple food	Tea and coffee	−0.09	−0.28
2101	Lunch	Rice	Total vegetables, tea and coffee, fish, meat, eggs	0.08	0.23
2102	Lunch	Rice	Total vegetables, tea and coffee, fish, meat	0.06	0.21
2103	Lunch	Rice	Total vegetables, tea and coffee, fish, eggs	0.12	0.21
2104	Lunch	Rice	Total vegetables, tea and coffee, fish, pulses	0.18	0.24
2111	Lunch	Rice	Total vegetables, meat, fish	0.08	0.20
2203	Lunch	Noodles	Tea and coffee	−0.23	−0.30
2401	Lunch	Rice	Tea and coffee	−0.25	−0.21
3102	Dinner	Rice	Total vegetables, tea and coffee, fish, pulses, fruit	0.13	0.24
3105	Dinner	Rice	Total vegetables, tea and coffee, fish, meat	−0.22	0.03
3107	Dinner	Rice	Total vegetables, tea and coffee, fish	−0.29	−0.06
3110	Dinner	Rice	Total vegetables, tea and coffee, meat	−0.28	−0.05
3115	Dinner	Rice	Total vegetables, fish	−0.21	−0.07
3116	Dinner	Rice	Total vegetables, meat, potatoes	−0.20	0.04
3117	Dinner	Rice	Total vegetables, meat	−0.28	−0.04
3118	Dinner	Rice	Total vegetables	−0.20	−0.04
3401	Dinner	Rice	Tea and coffee	−0.38	−0.24

^a^ Only data on the food combinations having Spearman correlation coefficients less than −0.20 or more than 0.20 with either HEI-2015 or NRF9.3 are shown.

## Supplementary Materials

The following are available online at https://www.mdpi.com/2072-6643/12/2/327/s1, Figure S1: Flow diagram of participants included in the present analysis, Figure S2: Flow diagram of the development process of the food combination database, Figure S3: An example of meal coding procedure, Figure S4: An example of the calculation of daily consumption frequency of food combinations (meal codes), based on an ad hoc computer algorithm for determining the food combinations each participant consumed, using information collected from the food combination questionnaire (FCQ), Figure S5: An example of the calculation of daily consumption frequency of accompanying foods, using the information collected from the food combination questionnaire (FCQ), Table S1: Food combination database used in this study, Table S2: Comparison of overall diet quality scores and overall dietary intakes assessed by FCQ and those assessed by BDHQ in 2233 Japanese adults aged 19–80 years, Table S3: Correlations among diet quality scores for each meal and overall diet quality scores in 2233 Japanese adults aged 19–80 years, Table S4: Food group intake in snacks in relation to diet quality scores for snacks in 2233 Japanese adults aged 19–80 years, Table S5: Food combinations (meal codes) in lunch in relation to diet quality scores for lunch in 2233 Japanese adults aged 19–80 years, Table S6: Food combinations (meal codes) in dinner in relation to diet quality scores for dinner in 2233 Japanese adults aged 19–80 years, Table S7: Food combinations (meal codes) in snacks in relation to diet quality scores for snacks in 2233 Japanese adults aged 19–80 years.
