# Supplementary material for: Food Combinations in Relation to the Quality of Overall Diet and Individual Meals in Japanese Adults: A Nationwide Study
Source: Nutrients. 2020 Jan 26;12(2):327. doi: 10.3390/nu12020327 (PMC7071262; doi:10.3390/nu12020327)
Supplement: Supplementary file 1 [file nutrients-12-00327-s001.pdf]

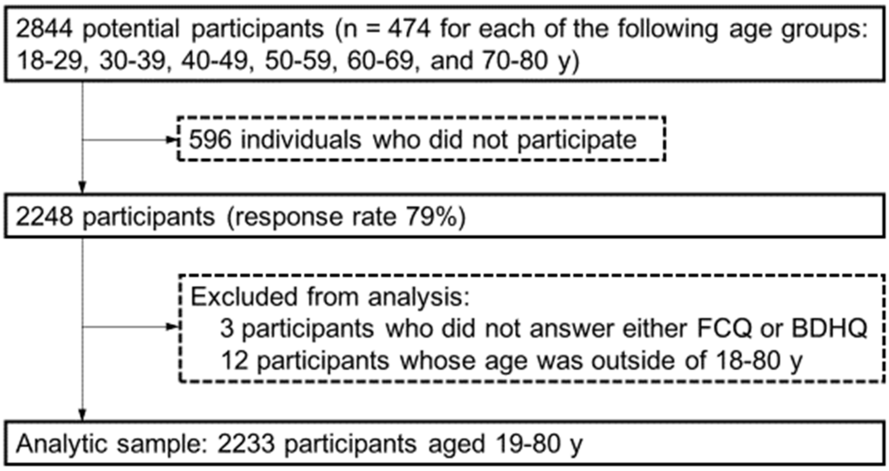

Figure S1: Flow diagram of participants included in the present analysis. BDHQ, brief diet history questionnaire; FCQ, food combination questionnaire.

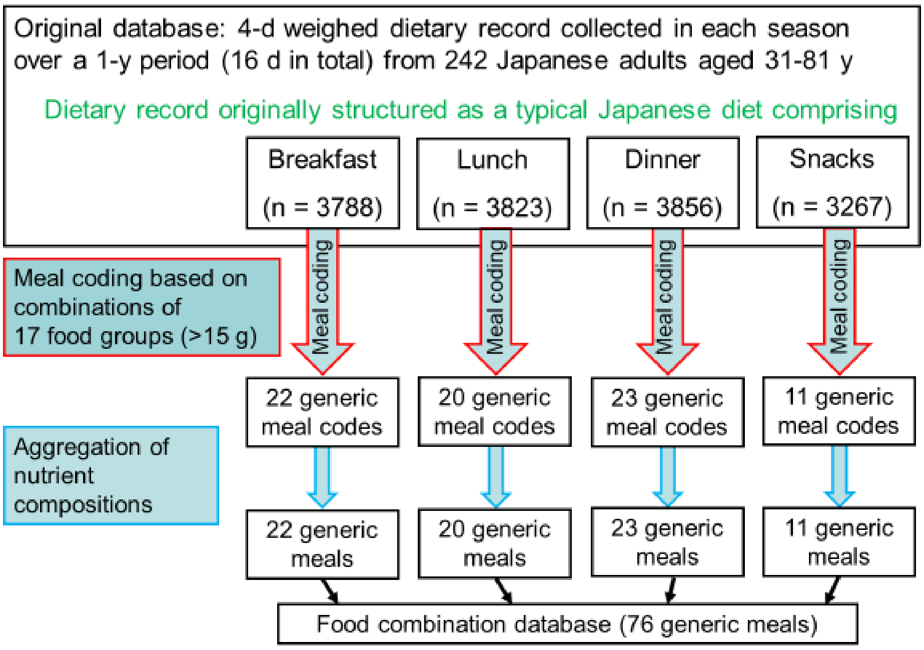

Figure S2: Flow diagram of the development process of the food combination database.

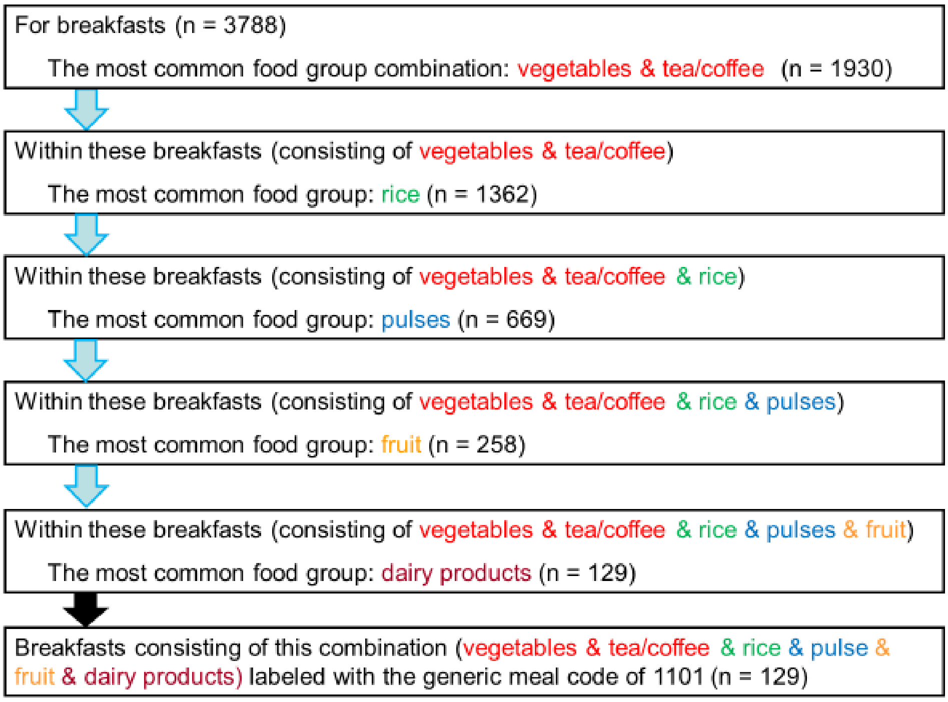

Figure S3: An example of meal coding procedure. For the breakfast meal type, the most commonly consumed combination of food groups at >15 g (an arbitrarily determined amount for consumption) was 'vegetables and tea and coffee' (n = 1930). Among all individual breakfasts comprising a combination of 'vegetables and tea and coffee', the most commonly consumed food group was 'rice' (n = 1362). For all individual breakfasts consisting of the 'vegetables, tea and coffee, and rice' combination, the most commonly consumed food group was 'pulses' (n = 669). For all individual breakfasts consisting of the 'vegetables, tea and coffee, rice, and pulses' combination, the most commonly consumed food group was 'fruit' (n = 258). For all individual breakfasts consisting of the 'vegetables, tea and coffee, rice, pulses, and fruit' combination, the most commonly consumed food group was 'dairy products' (n = 129). Based on this, we considered that the 'vegetables, tea and coffee, rice, pulses, fruit, and dairy products' combination represented a food group combination pattern and labeled it with the generic meal code of 1101. After categorizing breakfasts, which included combinations of 'vegetables and tea and coffee' (meal codes 1101-1112), we identified the next most commonly consumed food group combination ('bread and dairy products') and categorized it similarly (meal codes 1201-1204). We then repeated this process stepwise until arriving at the point at which the next most commonly consumed food group combination represented <2% of the total number of breakfasts (in this case, 'dairy products and tea and coffee'; meal code 1601). We then identified breakfasts, which consisted of not only a single food group, but which also accounted for >2% of the total number of breakfasts, and identified these as one category (in this case, 'tea and coffee'; meal code 1701). We repeated this process stepwise for the other meal types. Finally, we established 76 generic meals accounting for all meal types, after excluding 'all other combinations' for each meal. Each meal was then assigned a unique generic meal code.

Section 1. Answers in the FCQ (an example: rice as a staple food in breakfast)

Staple food	Accompanying food						
RIC	EGG	FSH	TVG	PLN	FRT	DRP	NNB
5 d/wk	Sometimes	Sometimes	Sometimes	Always	Never	Always	Always

Section 2. Food combination database characterized by possible answers for accompanying foods (only shown for meal codes including rice as a staple food in breakfast)

Meal code	RIC	EGG	FSH	TVG	PLN	FRT	DRP	NNB
1101	Always	Sometimes	Sometimes	Always	Always	Always	Always	Always
1102	Always	Sometimes	Sometimes	Always	Always	Always	Never	Always
1103	Always	Always	Sometimes	Always	Always	Never	Sometimes	Always
1104	Always	Never	Always	Always	Always	Never	Sometimes	Always
1105	Always	Never	Never	Always	Always	Never	Sometimes	Always
1106	Always	Always	Sometimes	Always	Never	Sometimes	Sometimes	Always
1107	Always	Never	Always	Always	Never	Sometimes	Sometimes	Always
1108	Always	Never	Never	Always	Never	Sometimes	Sometimes	Always
1301	Always	Sometimes	Sometimes	Always	Sometimes	Sometimes	Always	Never
1302	Always	Sometimes	Sometimes	Always	Sometimes	Sometimes	Never	Never
1401	Always	Sometimes	Sometimes	Never	Sometimes	Sometimes	Sometimes	Always

Section 3. Coding rule for each answer against food combination database

		Food combination database		
		Always	Sometimes	Never
Answer	Always	1	2	3
	Sometimes	7	8	9
	Never	6	5	4

Section 4. Determination of food combination (an example: rice as a staple food in breakfast)

Meal code	Code based on answer							A	B	C	D	Frequency (per d)
	EGG	FSH	TVG	PLN	FRT	DRP	NNB	Exact match (n of codes 1, 4, 8)	Partial match (n of codes 2, 5, 7, 9)	No match (n of codes 3, 6)	Coefficient (A × 2 + B − C × 2)	Frequency of staple food (5/7 here) × D ÷ Σ(D)
1101	8	8	7	1	6	1	1	5	1	1	9	0.0794
1102	8	8	7	1	6	3	1	4	1	2	5	0.0441
1103	7	8	7	1	4	2	1	4	3	0	11	0.0970
1104	9	7	7	1	4	2	1	3	4	0	10	0.0882
1105	9	9	7	1	4	2	1	3	4	0	10	0.0882
1106	7	8	7	3	5	2	1	2	4	1	6	0.0529
1107	9	7	7	3	5	2	1	1	5	1	5	0.0441
1108	9	9	7	3	5	2	1	1	5	1	5	0.0441
1301	8	8	7	2	5	1	3	3	3	1	7	0.0617
1302	8	8	7	2	5	3	3	2	3	2	3	0.0265
1401	8	8	9	2	5	2	1	3	4	0	10	0.0882

Figure S4: An example of the calculation of daily consumption frequency of food combinations (meal codes), based on an ad hoc computer algorithm for determining food combinations each participant consumed, using information collected from the food combination questionnaire (FCQ). First, based on the possible answers for accompanying foods ('always', 'sometimes', and 'never') in the FCQ (an example in Section 1), each of the food combinations (meal codes) was characterized in the same manner (Section 2). In this characterization, foods labeled as 'always' were those always included in the meal code (shown in bold in Supplemental Table 1), foods labeled as 'never' were those not included (always excluded) in the meal code (shown in shadow in Supplemental

Table 1), and foods labeled as 'sometimes' were all other foods. All the answers were then coded based on the coding rule (Section 3). A coefficient value was then calculated for each meal code (an example in Section 4); if the coefficient value was negative, a value of zero was assigned. Finally, the consumption frequency for each meal code was calculated based on the consumption frequency of the staple food weighted by the coefficient value as a proportion of the sum of coefficient values with the same staple food in each meal. DRP, dairy products; EGG, eggs; FRT, fruit; FSH, fish (including shellfish); NNB, tea and coffee (i.e., nonalcoholic and noncaloric beverages); PLN, pulses (including nuts); RIC, rice; TVG, total vegetables.

Section 1. Answers in the FCQ (an example: rice as a staple food in breakfast)

Staple food	Accompanying food						
RIC	EGG	FSH	TVG	PLN	FRT	DRP	NNB
5 d/wk	Sometimes	Sometimes	Sometimes	Always	Never	Always	Always

Section 2. Calculation of consumption frequency of accompanying foods (an example: rice as a staple food in breakfast)

		EGG	FSH	TVG	PLN	FRT	DRP	NNB
Factor based on the answer for relative consumption frequency	Always =1, sometimes = 0.5, never = 0	0.5	0.5	0.5	1	0	1	1
Frequency (per d)	Frequency of staple food (5/7 here) × factor	0.36	0.36	0.36	0.71	0	0.71	0.71

Figure S5: An example of the calculation of daily consumption frequency of accompanying foods, using the information collected from the food combination questionnaire (FCQ). For each staple food for each meal, the daily consumption frequency of accompanying foods was calculated as the daily consumption frequency of the corresponding staple food multiplied by a factor determined based on the answer for relative consumption frequency. DRP, dairy products; EGG, eggs; FRT, fruit; FSH, fish (including shellfish); NNB, tea and coffee (i.e., nonalcoholic and noncaloric beverages); PLN, pulses (including nuts); RIC, rice; TVG, total vegetables.

Table S1: Food combination database used in this study[a].

Meal code	Energy (kcal)	Meal composition (g of wet weight)[b]																			
		RIC	BRD	NDL	OGR	PTT	SGR[c]	PLN	TVG	FRT	FSH	MET	EGG	DRP	FOL[c]	CON	FVJ	ALB	SDK	NNB	SSN[c]
Breakfast																					
1101	650	**127**	3	2	3	10	3	**55**	**91**	**88**	17	4	16	**181**	2	2	0	1	1	**220**	73
1102	578	**146**	0	1	2	19	2	**56**	**125**	**87**	24	5	12	0	2	2	1	1	15	**205**	122
1103	581	**161**	1	0	5	7	1	**47**	**86**	1	11	6	**47**	42	2	1	0	0	1	**225**	88
1104	545	**154**	0	0	2	10	1	**61**	**96**	1	**44**	2	0	49	1	1	0	0	1	**210**	91
1105	491	**153**	0	1	5	8	1	**55**	**85**	1	4	6	1	48	1	1	0	0	2	**225**	79
1106	579	**147**	2	3	11	9	3	2	**103**	22	14	10	**49**	57	4	1	2	0	2	**235**	71
1107	536	**154**	1	5	7	10	2	3	**99**	20	**48**	6	1	48	2	1	1	0	3	**235**	65
1108	492	**145**	3	4	8	16	1	3	**99**	36	2	14	1	62	2	2	2	0	3	**229**	68
1109	550	0	**70**	0	1	3	6	6	**77**	42	4	9	**45**	**127**	7	1	18	0	1	**175**	17
1110	456	0	**67**	0	1	3	5	5	**84**	53	3	12	0	**133**	4	2	4	0	0	**186**	14
1111	404	0	**70**	0	0	3	4	4	**75**	29	4	8	24	3	6	3	8	0	1	**186**	24
1112	474	1	0	16	49	10	5	20	**105**	44	9	7	19	87	3	11	6	0	6	**228**	38
1201	446	0	**74**	0	0	1	4	2	2	**109**	1	2	10	**127**	4	3	2	0	9	**205**	10
1202	373	3	**71**	0	0	1	6	1	1	0	2	2	12	**93**	4	2	0	0	3	**217**	5
1203	558	4	**76**	0	1	0	3	5	**65**	43	3	11	28	**184**	4	0	9	0	35	0	14
1204	452	1	**72**	0	1	0	4	5	1	42	1	7	9	**190**	5	0	3	0	30	0	4
1301	581	**137**	0	2	2	9	2	20	**104**	39	20	7	23	**164**	2	1	1	1	6	0	68
1302	495	**156**	2	2	3	7	1	24	**98**	26	20	8	14	0	2	1	2	0	12	0	85
1401	428	**143**	1	2	4	7	2	18	5	27	9	5	21	40	1	0	2	0	2	227	49
1501	337	0	**72**	0	0	1	4	2	2	24	0	7	14	2	5	1	1	0	3	**232**	6
1601	327	0	0	0	5	2	10	1	0	38	0	4	4	**139**	2	28	1	0	7	**304**	1
1701	27	0	0	0	0	0	3	0	0	0	0	0	0	1	0	0	0	0	0	**282**	0
Lunch																					
2101	845	**210**	1	7	11	10	3	10	**84**	20	**48**	**45**	**44**	6	10	2	1	6	3	**249**	45
2102	737	**192**	1	11	7	13	2	12	**97**	25	**52**	**43**	2	8	6	3	1	12	4	**229**	47
2103	671	**183**	0	6	10	7	3	9	**90**	17	**60**	2	**42**	14	5	2	2	5	2	**221**	53
2104	649	**171**	0	6	6	15	3	**56**	**102**	23	**69**	2	2	6	3	3	1	20	0	**212**	69
2105	608	**178**	0	14	7	10	2	1	**101**	23	**69**	2	2	8	4	2	1	17	1	**211**	52
2106	733	**183**	2	13	10	11	2	12	**93**	17	2	**49**	**46**	8	7	3	2	1	1	**233**	55
2107	717	**180**	0	11	7	**51**	3	14	**112**	16	1	**58**	1	8	6	3	1	1	2	**216**	73
2108	665	**170**	0	24	8	1	2	16	**118**	26	1	**54**	2	8	7	3	0	3	1	**233**	67

Table S1 (continued)

Meal code	Energy (kcal)	Meal composition (g of wet weight)[b]																			
		RIC	BRD	NDL	OGR	PTT	SGR[c]	PLN	TVG	FRT	FSH	MET	EGG	DRP	FOL[c]	CON	FVJ	ALB	SDK	NNB	SSN[c]
2109	548	**157**	0	16	6	5	3	**68**	**106**	28	3	2	15	6	2	0	1	1	1	**193**	76
2110	503	**157**	3	31	4	13	2	2	**105**	19	2	3	14	12	3	3	3	1	2	**218**	43
2111	840	**200**	0	16	8	12	2	12	**113**	13	**55**	**47**	20	7	7	1	4	44	4	0	74
2112	755	**181**	1	26	8	20	2	17	**111**	19	2	**54**	13	19	7	4	3	15	9	0	72
2113	634	**166**	1	13	6	13	2	28	**104**	26	**60**	2	14	23	4	1	2	20	11	0	74
2114	493	**143**	1	26	5	14	2	15	**104**	19	2	3	20	11	4	1	0	11	11	0	63
2201	638	0	3	**193**	4	5	1	5	**109**	31	7	**42**	12	18	6	4	1	6	3	**228**	80
2202	556	0	2	**217**	4	10	2	15	**92**	23	22	2	12	4	4	0	0	5	1	**204**	89
2203	531	49	0	**194**	5	1	2	6	6	10	13	9	10	12	2	2	0	2	2	**221**	77
2301	727	0	39	73	24	23	3	5	**88**	24	10	**52**	12	54	8	9	5	33	30	80	66
2401	557	**184**	1	0	5	6	2	16	6	28	31	15	17	7	3	4	2	0	1	**258**	29
2501	639	1	**83**	4	2	4	13	13	42	50	4	7	14	**208**	7	6	7	0	9	81	11
Dinner																					
3101	952	**163**	1	4	10	28	4	**81**	**164**	33	**81**	**54**	14	5	7	1	1	108	1	**216**	104
3102	815	**160**	0	3	5	13	5	**74**	**151**	**95**	**97**	1	6	17	3	3	0	73	0	**221**	79
3103	780	**160**	0	9	4	16	4	**84**	**156**	1	**91**	1	7	8	4	4	1	83	1	**195**	97
3104	933	**163**	1	4	7	**79**	4	1	**172**	40	**71**	**67**	7	10	7	4	0	102	1	**222**	77
3105	858	**154**	1	14	11	1	2	1	**141**	32	**74**	**71**	12	9	7	5	2	83	4	**225**	60
3106	686	**146**	0	5	6	25	3	3	**159**	**103**	**94**	1	8	13	4	3	0	17	0	**238**	57
3107	735	**176**	1	8	7	13	3	2	**140**	1	**100**	1	13	10	4	5	1	84	2	**227**	66
3108	839	**161**	2	11	6	**69**	4	29	**158**	37	2	**74**	9	14	7	4	2	35	0	**215**	73
3109	819	**156**	0	4	10	1	3	**79**	**168**	21	2	**82**	10	8	7	2	1	44	3	**228**	83
3110	801	**164**	1	15	14	1	1	2	**168**	26	2	**87**	9	16	7	4	1	49	0	**234**	49
3111	556	**149**	0	16	7	24	2	33	**153**	22	3	4	13	14	5	2	2	29	1	**196**	59
3112	1131	**161**	0	11	9	28	3	30	**164**	22	**89**	**64**	10	15	7	3	3	**433**	9	0	84
3113	955	**156**	0	8	9	20	4	30	**152**	39	**106**	1	11	14	6	4	3	**397**	2	0	78
3114	726	**156**	0	5	8	26	3	**69**	**128**	18	**75**	15	9	26	5	1	1	1	3	0	85
3115	709	**167**	0	15	7	24	3	2	**143**	26	**71**	24	10	21	5	1	5	1	3	0	74
3116	887	**169**	0	6	5	**63**	2	14	**161**	15	2	**77**	9	22	7	4	4	137	4	0	77
3117	914	**158**	0	8	10	2	2	23	**166**	27	1	**91**	9	23	8	3	3	216	12	0	69
3118	567	**135**	5	16	8	24	2	50	**156**	25	2	3	11	23	3	0	3	69	0	0	74
3201	1007	0	4	51	20	28	3	26	**162**	23	**90**	**71**	12	18	7	1	0	**474**	9	72	92

Table S1 (continued)

Meal code	Energy (kcal)	Meal composition (g of wet weight)[b]																			
		RIC	BRD	NDL	OGR	PTT	SGR[c]	PLN	TVG	FRT	FSH	MET	EGG	DRP	FOL[c]	CON	FVJ	ALB	SDK	NNB	SSN[c]
3202	1127	0	8	73	28	32	2	28	**185**	28	1	**113**	9	18	11	7	1	**496**	4	58	59
3203	650	0	6	102	24	22	2	26	**154**	28	21	**56**	8	23	6	5	9	1	17	141	76
3301	719	0	3	83	22	19	2	31	**137**	24	**90**	2	10	18	5	4	1	233	2	95	70
3401	678	**168**	0	30	3	13	2	31	6	20	60	31	10	7	2	3	3	51	5	**212**	50
Snacks																					
4101	464	2	3	1	3	3	5	4	4	29	1	1	1	**133**	0	**72**	3	19	29	**397**	2
4102	375	2	3	1	2	2	3	5	9	**111**	1	0	0	1	1	**69**	4	18	19	**379**	2
4103	284	0	1	1	1	1	1	3	2	0	1	1	0	1	0	**67**	3	30	30	**375**	1
4201	271	1	4	1	4	3	8	3	8	32	1	1	1	**130**	1	2	7	27	33	**353**	2
4301	171	2	3	1	4	2	3	7	10	**120**	1	1	1	1	0	2	7	14	14	**341**	2
4401	177	4	3	2	0	1	2	1	3	0	2	1	0	1	0	2	5	50	**202**	**368**	4
4501	422	1	3	0	0	2	1	5	1	26	0	0	1	**168**	0	**68**	6	5	31	0	0
4601	389	1	1	4	2	1	2	4	7	0	5	3	0	1	1	1	0	**506**	0	**416**	6
4701	33	0	0	0	0	0	2	0	0	0	0	0	0	2	0	1	0	0	0	**385**	0
4702	209	0	0	0	0	0	0	0	0	0	0	0	0	0	0	**63**	0	0	0	0	0
4703	123	0	0	0	0	0	0	0	0	0	0	0	0	0	0	1	0	0	**265**	0	0

[a] The food combination database (meal coding system) was developed according to the 'frequent item sets' data-mining method, using 16-day weighed dietary record data collected from 242 Japanese adults aged 31-81 years, which included 3788 breakfasts, 3823 lunches, 3856 dinners, and 3267 snacks. [b] Abbreviations for food groups are as follows: RIC, rice; BRD, bread; NDL, noodles; OGR, other grains; PTT, potatoes; SGR, sugars; PLN, pulses (including nuts); TVG, total vegetables; FRT, fruit; FSH, fish (including shellfish), MET, meat; EGG, eggs; DRP, dairy products; FOL, fats and oils; CON, confectioneries; FVJ, fruit and vegetable juice; ALB, alcoholic beverages; SDK, soft drinks; NNB, tea and coffee (i.e., nonalcoholic and noncaloric beverages); SSN, seasonings. Food groups which were always included in the meal code are shown in bold while food groups not included (always excluded) in the meal code are shown in shadow. [c] Not considered in development of the meal coding system.

Table S2: Comparison of overall diet quality scores and overall dietary intakes assessed by FCQ and those assessed by BDHQ in 2233 Japanese adults aged 19-80 years[a].

	FCQ		BDHQ		Spearman correlation between FCQ and BDHQ		
	Mean	SD	Mean	SD	Total	Men[b]	Women[c]
HEI-2015[d]	53.3	2.7	56.4	6.6	0.49	0.50	0.45
Total fruits[e]	1.6	0.5	2.3	1.6	0.55	0.52	0.53
Whole fruits[e]	3.1	1.0	3.0	1.8	0.56	0.55	0.52
Total vegetables[e]	4.7	0.6	4.6	0.8	0.37	0.40	0.32
Greens and beans[e]	3.7	0.9	3.7	1.5	0.34	0.37	0.30
Whole grains[f]	0.9	0.2	0.6	0.7	0.21	0.22	0.16
Dairy[f]	2.2	1.0	2.2	1.6	0.60	0.63	0.55
Total protein foods[e]	4.6	0.4	4.9	0.3	0.19	0.21	0.18
Seafood and plant proteins[e]	5.0	0.2	4.9	0.4	0.18	0.22	0.12
Fatty acids[f]	7.7	1.5	8.2	2.1	0.32	0.33	0.27
Refined grains[f]	0.1	0.4	2.2	2.8	0.34	0.34	0.34
Sodium[f]	0.0	0.0	0.5	1.3	0.04	0.06	0.02
Added sugars[f]	9.9	0.2	9.7	0.9	0.30	0.34	0.27
Saturated fats[f]	9.8	0.5	9.5	1.1	0.30	0.27	0.30
NRF9.3[g]	709	56	718	97	0.48	0.52	0.54
Protein[h]	100	0	100	1	0.24	---[i]	0.38
Dietary fiber[h]	81	11	77	17	0.36	0.39	0.38
Vitamin A[h]	80	12	87	18	0.21	0.20	0.23
Vitamin C[h]	94	10	94	14	0.26	0.25	0.31
Vitamin D[h]	96	9	99	7	0.16	0.23	0.16
Calcium[h]	80	12	86	17	0.46	0.50	0.46
Iron[h]	93	12	95	12	0.79	0.09[j]	0.82
Potassium[h]	93	8	95	10	0.30	0.31	0.35
Magnesium[h]	94	6	92	11	0.35	0.40	0.34
Added sugars	15	17	20	43	0.37	0.39	0.34
Saturated fats	7	10	12	17	0.41	0.38	0.39
Sodium	80	21	75	35	0.38	0.30	0.20
Food group (g of wet weight per 1000 kcal)							
Rice	186.1	51.7	147.9	65.6	0.61	0.59	0.62
Bread	26.0	17.7	18.9	15.3	0.75	0.76	0.73
Noodles	42.6	21.7	34.5	23.4	0.52	0.51	0.52
Other grains	13.9	4.8	NA	NA	---	---	---
Potatoes	16.1	4.2	25.9	19.5	0.14	0.17	0.11
Sugars	7.2	1.7	2.9	2.3	0.07	0.08	0.03
Pulses (including nuts)	24.9	6.4	38.8	25.4	0.45	0.46	0.45
Total vegetables	131.9	38.5	151.5	75.4	0.45	0.47	0.42

Table S2 (continued)

	FCQ		BDHQ		Spearman correlation between FCQ and BDHQ		
	Mean	SD	Mean	SD	Total	Men[b]	Women[c]
Fruit	36.1	11.6	49.4	43.2	0.58	0.56	0.55
Fish (including shellfish)	28.0	6.7	46.0	24.3	0.31	0.31	0.32
Meat	27.7	7.2	46.4	19.2	0.30	0.34	0.27
Eggs	16.8	3.3	23.6	14.7	0.42	0.41	0.42
Dairy products	59.6	29.4	60.8	52.8	0.60	0.63	0.55
Fats and oils	8.6	1.0	10.6	4.0	0.19	0.19	0.19
Confectioneries	12.5	5.3	32.9	22.7	0.53	0.53	0.48
Fruit and vegetable juice	5.7	1.5	21.1	40.4	0.12	0.14	0.10
Alcoholic beverages	40.0	44.9	63.9	118.7	0.82	0.82	0.77
Soft drinks	13.4	6.6	24.4	47.8	0.36	0.36	0.38
Tea and coffee (i.e., nonalcoholic and noncaloric beverages	363.4	104.9	318.7	196.8	0.34	0.38	0.28
Seasonings	101.6	14.8	84.3	49.4	0.22	0.21	0.22
Macronutrient (% of energy)							
Protein	13.2	0.8	16.0	2.9	0.42	0.41	0.40
Fat	26.0	2.2	28.1	5.5	0.42	0.37	0.40
Saturated fats	7.2	1.0	7.3	1.8	0.45	0.42	0.42
Monounsaturated fats	9.7	0.8	10.1	2.2	0.39	0.36	0.38
Polyunsaturated fats	6.1	0.3	7.0	1.5	0.27	0.22	0.31
Carbohydrate	57.6	3.3	50.9	7.8	0.49	0.50	0.47
Alcohol	1.9	2.2	3.8	6.6	0.82	0.84	0.77
Energy (kcal/d)	1655	203	1840	532	0.40	0.45	0.46

BDHQ, brief diet history questionnaire; FCQ, food combination questionnaire; HEI-2015, Healthy Eating Index-2015; NA, not available; NRF9.3, Nutrient-Rich Food Index 9.3. [a] A higher score indicates a higher diet quality, except for added sugars, saturated fats, and sodium components in NRF9.3, for which a higher score indicates an unfavorable dietary intake (i.e., higher intakes of added sugars, saturated fats, and sodium). [b] Spearman correlations calculated based on men only (n = 1070). [c] Spearman correlations calculated based on women only (n = 1163). [d] Calculated as the sum of all component scores. A maximum score is 100. [e] A maximum score is 5. [f] A maximum score is 10. [g] Calculated as the sum of scores for nine nutrients to encourage (i.e., protein, dietary fiber, vitamins A, C, and D, calcium, iron, potassium, and magnesium) minus the sum of scores for three nutrients to limit (i.e., added sugar, saturated fats, and sodium). A maximum score is 900. [h] A maximum score is 100. [i] Not calculated because all men had a maximum score. [j] Note that almost all men had a maximum score.

Table S3: Correlations among diet quality scores for each meal and overall diet quality scores in 2233 Japanese adults aged 19-80 years[a].

	HEI-2015 Breakfast	HEI-2015 Lunch	HEI-2015 Dinner	HEI-2015 Snacks	HEI-2015 Overall diet	NRF9.3 Breakfast	NRF9.3 Lunch	NRF9.3 Dinner	NRF9.3 Snacks
HEI-2015									
Lunch	0.21	1							
Dinner	0.16	0.18	1						
Snacks	0.17	0.11	0.12	1					
Overall diet	0.71	0.49	0.52	0.29	1				
NRF9.3									
Breakfast	0.82	0.23	0.10	0.18	0.55	1			
Lunch	0.20	0.75	0.15	0.08	0.39	0.28	1		
Dinner	0.21	0.36	0.17	0.14	0.48	0.28	0.38	1	
Snacks	0.14	0.10	0.10	0.77	0.22	0.19	0.20	0.16	1
Overall diet	0.50	0.53	0.25	0.22	0.67	0.62	0.67	0.71	0.33

HEI-2015, Healthy Eating Index-2015; NRF9.3, Nutrient-Rich Food Index 9.3. [a] Values are Spearman correlation coefficients. A higher score indicates a higher diet quality.

Table S4: Food group intake in snacks in relation to diet quality scores for snacks in 2233 Japanese adults aged 19-80 years[a].

	Intake (g of wet weight per 1000 kcal of energy from snacks)			r with	r with
Food group	Median	P25	P75	HEI-2015	NRF9.3
Total vegetables	32.6	26.2	36.3	0.60	0.66
Fruit	78.6	0	97.5	0.69	0.43
Dairy products	114.9	0	153.7	0.17	0.22
Confectioneries	120.9	104.8	155.1	-0.58	-0.41
Fruit and vegetable juice	26.4	21.5	27.2	0.60	0.50
Soft drinks	95.5	0	134.2	-0.10	-0.35
Tea and coffee (i.e., nonalcoholic and noncaloric beverages)	1879.9	1332.5	2254.7	0.27	0.46

HEI-2015, Healthy Eating Index-2015; NRF9.3, Nutrient-Rich Food Index 9.3; P25, 25th percentile; P75, 75th percentile. [a] Spearman correlation coefficients between intakes of food groups in snacks and HEI-2015 and NRF9.3 of snacks were calculated. For both HEI-2015 and NRF9.3, a higher score indicates a higher diet quality. Only data on the food groups whose median value was >25 g/1000 kcal are shown.

Table S5: Food combinations (meal codes) in lunch in relation to diet quality scores for lunch in 2233 Japanese adults aged 19-80 years[a].

	Food group included		Intake (% of total energy)				
Meal code	Staple food	Accompanying food[b]	Median	P25	P75	r with HEI-2015	r with NRF9.3
2101	Rice	Total vegetables, tea and coffee, fish, meat, eggs	1.96	1.22	2.49	0.36	0.48
2102	Rice	Total vegetables, tea and coffee, fish, meat	1.72	1.14	2.15	0.32	0.43
2103	Rice	Total vegetables, tea and coffee, fish, eggs	1.47	0.94	1.87	0.37	0.44
2104	Rice	Total vegetables, tea and coffee, fish, pulses	1.19	0.73	1.60	0.46	0.46
2105	Rice	Total vegetables, tea and coffee, fish	1.21	0.81	1.53	0.19	0.25
2106	Rice	Total vegetables, tea and coffee, meat, eggs	1.72	1.16	2.17	0.18	0.31
2107	Rice	Total vegetables, tea and coffee, meat, potatoes	1.46	0.95	1.84	0.23	0.34
2108	Rice	Total vegetables, tea and coffee, meat	1.48	1.09	1.87	0.02	0.14
2109	Rice	Total vegetables, tea and coffee, pulses	1.21	0.81	1.53	0.25	0.28
2110	Rice	Total vegetables, tea and coffee	1.18	0.84	1.46	-0.02	0.06
2111	Rice	Total vegetables, meat, fish	1.44	0.89	1.86	0.35	0.43
2112	Rice	Total vegetables, meat	1.34	0.97	1.73	0.12	0.21
2113	Rice	Total vegetables, fish	1.04	0.64	1.34	0.35	0.36
2114	Rice	Total vegetables	0.84	0.59	1.07	0.09	0.11
2201	Noodles	Tea and coffee, total vegetables, meat	1.13	0	2.28	0.30	0.06
2202	Noodles	Tea and coffee, total vegetables	1.43	0	2.69	0.18	-0.12
2203	Noodles	Tea and coffee	1.57	0.43	3.25	-0.28	-0.41
2301	No staple food	Total vegetables, meat	0	0	0	-0.02	0.00
2401	Rice	Tea and coffee	1.39	1.04	1.76	-0.24	-0.17
2501	Bread	Dairy products	0	0	5.05	-0.13	-0.07

HEI-2015, Healthy Eating Index-2015; NRF9.3, Nutrient-Rich Food Index 9.3; P25, 25th percentile; P75, 75th percentile. [a] Spearman correlation coefficients between intakes of meal codes in lunch (as assessed by percentage of total energy intake) and the quality of lunch as assessed by the HEI-2015 and NRF9.3 were calculated. For both HEI-2015 and NRF9.3, a higher score indicates a higher diet quality. [b] 'Tea and coffee' consisting of nonalcoholic and noncaloric beverages; 'fish' including shellfish; 'pulses' including nuts.

Table S6: Food combinations (meal codes) in dinner in relation to diet quality scores for dinner in 2233 Japanese adults aged 19-80 years[a].

	Food group included		Intake (% of total energy)				
Meal code	Staple food	Accompanying food[b]	Median	P25	P75	r with HEI-2015	r with NRF9.3
3101	Rice	Total vegetables, tea and coffee, fish, pulses, meat	2.34	1.55	2.84	-0.57	0.37
3102	Rice	Total vegetables, tea and coffee, fish, pulses, fruit	1.57	0.93	2.06	-0.31	0.47
3103	Rice	Total vegetables, tea and coffee, fish, pulses	1.64	1.08	2.06	-0.69	0.29
3104	Rice	Total vegetables, tea and coffee, fish, meat, potatoes	1.91	1.19	2.38	-0.58	0.23
3105	Rice	Total vegetables, tea and coffee, fish, meat	1.79	1.22	2.19	-0.63	0.16
3106	Rice	Total vegetables, tea and coffee, fish, fruit	1.23	0.72	1.55	-0.35	0.29
3107	Rice	Total vegetables, tea and coffee, fish	1.42	0.87	1.82	-0.76	0.09
3108	Rice	Total vegetables, tea and coffee, meat, potatoes	1.96	1.28	2.37	-0.62	0.27
3109	Rice	Total vegetables, tea and coffee, meat, pulses	1.82	1.22	2.19	-0.61	0.30
3110	Rice	Total vegetables, tea and coffee, meat	1.62	1.08	2.01	-0.66	0.09
3111	Rice	Total vegetables, tea and coffee	1.31	0.88	1.63	-0.64	0.22
3112	Rice	Total vegetables, fish, alcoholic beverages, meat	1.59	1.10	2.25	-0.51	0.28
3113	Rice	Total vegetables, fish, alcoholic beverages	1.22	0.74	1.74	-0.47	0.28
3114	Rice	Total vegetables, fish, pulses	1.38	0.89	1.76	-0.55	0.27
3115	Rice	Total vegetables, fish	1.23	0.79	1.62	-0.62	0.06
3116	Rice	Total vegetables, meat, potatoes	1.48	0.96	1.78	-0.60	0.21
3117	Rice	Total vegetables, meat	1.56	1.08	1.89	-0.66	0.12
3118	Rice	Total vegetables	1.00	0.62	1.21	-0.61	0.15
3201	No staple food	Total vegetables, meat, alcoholic beverages, fish	0.64	0	2.42	0.68	-0.16
3202	No staple food	Total vegetables, meat, alcoholic beverages	1.46	0	3.93	0.66	-0.23
3203	No staple food	Total vegetables, meat	2.35	0	6.34	0.65	-0.29
3301	No staple food	Total vegetables, fish	1.08	0	3.02	0.69	-0.25
3401	Rice	Tea and coffee	1.67	1.18	1.99	-0.77	-0.14

HEI-2015, Healthy Eating Index-2015; NRF9.3, Nutrient-Rich Food Index 9.3; P25, 25th percentile; P75, 75th percentile. [a] Spearman correlation coefficients between intakes of meal codes in dinner (as assessed by percentage of total energy intake) and the quality of dinner as assessed by the HEI-2015 and NRF9.3 were calculated. For both HEI-2015 and NRF9.3, a higher score indicates a higher diet quality. [b] 'Tea and coffee' consisting of nonalcoholic and noncaloric beverages; 'fish' including shellfish; 'pulses' including nuts.

Table S7: Food combinations (meal codes) in snacks in relation to diet quality scores for snacks in 2233 Japanese adults aged 19-80 years[a].

	Food group included		Intake (% of total energy)				
Meal code	Staple food	Accompanying food[b]	Median	P25	P75	r with HEI-2015	r with NRF9.3
4101	No staple food	Confectioneries, tea and coffee, dairy products	1.83	0.98	2.57	0.04	0.00
4102	No staple food	Confectioneries, tea and coffee, fruit	1.41	0.75	1.90	0.13	0.02
4103	No staple food	Confectioneries, tea and coffee	1.23	0.76	1.61	-0.22	-0.16
4201	No staple food	Dairy products, tea and coffee	0.95	0.48	1.36	0.39	0.34
4301	No staple food	Fruit, tea and coffee	0.56	0.31	0.79	0.57	0.43
4401	No staple food	Soft drinks, tea and coffee	0.48	0.26	0.69	0.06	0.04
4501	No staple food	Confectioneries, dairy products	0.93	0.38	1.51	-0.01	-0.12
4601	No staple food	Alcoholic beverages, tea and coffee	0.81	0.35	1.28	0.28	0.48
4701	No staple food	Tea and coffee	0.12	0.07	0.16	0.09	0.24
4702	No staple food	Confectioneries	0.51	0.27	0.80	-0.30	-0.17
4703	No staple food	Soft drinks	0.16	0.03	0.30	-0.08	-0.15

HEI-2015, Healthy Eating Index-2015; NRF9.3, Nutrient-Rich Food Index 9.3; P25, 25th percentile; P75, 75th percentile. [a] Spearman correlation coefficients between intakes of meal codes in snacks (as assessed by percentage of total energy intake) and the quality of snacks as assessed by the HEI-2015 and NRF9.3 were calculated. For both HEI-2015 and NRF9.3, a higher score indicates a higher diet quality. [b] 'Tea and coffee' consisting of nonalcoholic and noncaloric beverages.
